# A Method for Recording the Bioelectrical Activity of Neural Axons upon Stimulation with Short Pulses of Infrared Laser Radiation

**DOI:** 10.17691/stm2020.12.6.03

**Published:** 2020-12-28

**Authors:** Ya.I. Pigareva, O.O. Antipova, V.N. Kolpakov, O.V. Martynova, A.A. Popova, I.V. Mukhina, A.S. Pimashkin, V.A. Es’kin

**Affiliations:** Junior Researcher, Laboratory of Neuro-engineering, Research Institute of Neurosciences; National Research Lobachevsky State University of Nizhni Novgorod, 23 Prospekt Gagarina, Nizhny Novgorod, 603950, Russia;; Assistant, Laboratory of Neuro-engineering, Research Institute of Neurosciences; National Research Lobachevsky State University of Nizhni Novgorod, 23 Prospekt Gagarina, Nizhny Novgorod, 603950, Russia;; Junior Researcher, Laboratory of Neuro-engineering, Research Institute of Neurosciences; National Research Lobachevsky State University of Nizhni Novgorod, 23 Prospekt Gagarina, Nizhny Novgorod, 603950, Russia;; Engineer, Department of Electrodynamics, Faculty of Radiophysics; National Research Lobachevsky State University of Nizhni Novgorod, 23 Prospekt Gagarina, Nizhny Novgorod, 603950, Russia;; PhD Student, Department of Electrodynamics, Faculty of Radiophysics; National Research Lobachevsky State University of Nizhni Novgorod, 23 Prospekt Gagarina, Nizhny Novgorod, 603950, Russia;; Professor, Head of the Central Research Laboratory; Privolzhsky Research Medical University, 10/1 Minin and Pozharsky Square, Nizhny Novgorod, 603005, Russia Head of the Department of Normal Physiology named after N.Y. Belenkov; Privolzhsky Research Medical University, 10/1 Minin and Pozharsky Square, Nizhny Novgorod, 603005, Russia Professor, Department of Neurotechnology, Institute of Biology and Biomedicine; National Research Lobachevsky State University of Nizhni Novgorod, 23 Prospekt Gagarina, Nizhny Novgorod, 603950, Russia;; Associate Professor, Department of Neurotechnology; National Research Lobachevsky State University of Nizhni Novgorod, 23 Prospekt Gagarina, Nizhny Novgorod, 603950, Russia; Researcher, Laboratory of Neuro-engineering, Research Institute of Neurosciences; National Research Lobachevsky State University of Nizhni Novgorod, 23 Prospekt Gagarina, Nizhny Novgorod, 603950, Russia;; Associate Professor, Department of Electrodynamics, Faculty of Radiophysics National Research Lobachevsky State University of Nizhni Novgorod, 23 Prospekt Gagarina, Nizhny Novgorod, 603950, Russia;

**Keywords:** microelectrode array, extracellular electrophysiology, bioelectric activity of neurons, culture of hippocampal neurons, optical stimulation of neurons, IR radiation, microfluidics

## Abstract

**The aim of the study** was to develop a method for long-term non-invasive recording of the bioelectrical activity induced in isolated neuronal axons irradiated with short infrared (IR) pulses and to study the effect of radiation on the occurrence of action potentials in axons of a neuron culture *in vitro*.

**Materials and Methods.:**

Hippocampal cells of mouse embryos (E18) were cultured in microfluidic chips made of polydimethylsiloxane and containing microchannels for axonal growth at a distance of up to 800 μm. We studied the electrophysiological activity of a neuronal culture induced by pulses of focused laser radiation in the IR range (1907 and 2095 nm). The electrophysiological activity of the neuronal culture was recorded using a multichannel recording system (Multi Channel Systems, Germany).

**Results.:**

The developed microfluidic chip and the optical stimulation system combined with the multichannel registration system made it possible to non-invasively record the action potentials caused by pulsed IR radiation in isolated neuronal axons *in vitro*. The propagation of action potentials in axons was detected using extracellular microelectrodes when the cells were irradiated with a laser at a wavelength of 1907 nm with a radiation power of 0.2–0.5 W for pulses with a duration of 6 ms and 0.5 W for pulses with a duration of 10 ms. It was shown that the radiation power positively correlated with the occurrence rate of axonal response. Moreover, the probability of a response evoked by optical stimulation increased at short optical pulses. In addition, we found that more responses could be evoked by irradiating the neuronal cell culture itself rather than the axon-containing microchannels.

**Conclusion.:**

The developed method makes it possible to isolate the axons growing from cultured neurons into a microfluidic chip, stimulate the neurons with infrared radiation, and non-invasively record the axonal spiking. The proposed approach allowed us to study the characteristics of neuronal responses in cell cultures over a long (weeks) period of time. The method can be used both in fundamental research into the brain signaling system and in the development of a non-invasive neuro-interface.

## Introduction

Perception of information about the external world occurs with the help of sensory systems, in which receptors convert physicochemical signals into bioelectric impulses of nerve fibers for further transmission to the brain. Trauma, progressive neurological diseases, or age-related changes can lead to disruption of pathways involved in perceiving and transmitting the information. Restoration of lost sensory functions is possible through the use of invasive sensory implants. For example, cochlear, vestibular, and retinal implants are successfully used in medicine; those are designed to cause an electrical stimulation of nerve fibers by means of implanted microelectrodes. However, this approach has a number of disadvantages: with prolonged exposure, the accuracy/sensitivity of the cell membrane to stimulation decreases; in addition, implantation of microelectrodes can result in glial scarring.

Currently, methods for non-invasive excitation of sensory neurons with electromagnetic radiation of the optical or infrared (IR) wave ranges are being developed. A number of advantages make this approach promising: namely, the absence of direct contact between the radiation source and the tissue, the high spatial resolution, and the absence of electrochemical interaction with the tissue.

Earlier experiments showed that infrared laser radiation at a wavelength range of 1200–2200 nm could either excite or suppress neuronal activity. Thus, exposure of artificial phospholipid bilayers, mammalian cells, or oocytes to IR radiation causes depolarization of these membranes [1]. IR laser radiation focused on spiral and vestibular ganglia *in vitro* leads to the generation of action potentials [2]. Cayce et al. [3, 4] showed that pulsed IR radiation had the ability to either excite or suppress spontaneous neural activity in rodent and primate brains. Infrared nerve stimulation was first demonstrated in the sciatic nerve of a rabbit [5] and then investigated for potential clinical applications with the peripheral nervous system [6, 7], the cochlea [8], and the heart [9, 10]. Some mechanisms of this biological activity are discussed; among them, changes in the membrane electrical capacity due to local heating of the surrounding fluid upon absorption of infrared radiation [1, 11], and the mediating role of temperature-sensitive receptors [12, 13]. The search for the optimal irradiation conditions and the study of cellular mechanisms *in vivo* is complicated by the sophisticated structure of neural networks in the brain. Individual neurons are hardly accessible for experimentation *in vivo* for the same reason. Therefore, simplified *in vitro* models based on dissociated neuronal cells cultured on microelectrode arrays (MEA) are commonly used. Under the favorable growth conditions, these cell cultures remain viable for more than a month.

In MEA — monolayer neuronal cultures, IR radiation suppressed the neuron activity [14]. Generation of action potentials was detected at a distance of up to 200 μm from the focus of IR irradiation [15]. Until now, however, the exposure to IR was studied in homogeneous neuronal cultures; it was, therefore, difficult to identify the activity propagating along the axons. Also, the distance at which the evoked action potential was detected did not exceed 200 μm.

**The aim of this study** was to develop a method for long-term non-invasive recording of the evoked bioelectric activity in isolated neuronal axons when stimulated by short pulses of IR radiation. Using constructs made of polydimethylsiloxane (PDMS), we separated bundles of axons in microchannels with a length of 800 μm; then, using electrophysiological methods, we studied the parameters of infrared radiation capable of evoking responses in isolated axons.

## Materials and Methods

### Crafting of microfluidic chips.

**Figure 1:**
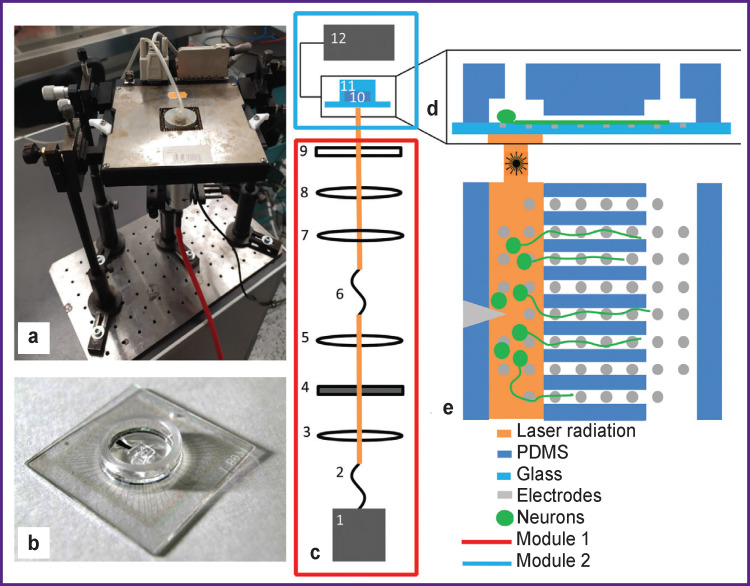
Setup for optical stimulation and recording of bioelectric activity in neuronal axons: (a) connector of the multichannel registration system with the installed MEA; (b) MEA combined with a microfluidic chip; (c) schematic representation of the entire system: (*1*) fiber laser; (*2*), (*6*) fiber optic; (*3*), (*5*), (*7*), (*8*) round-shaped collecting lenses; (*4*) shutter; (*9*) cylindrical collecting lens; (*10*) microfluidic chip; (*11*) MEA; (*12*) multi-channel recording system; (d) and (e) drawings of a microfluidic chip on the MEA, side and top views

 Microfluidic chips were made by pouring liquid PDMS onto a master mold followed by its solidifying. The technique for making microfluidic chips was described earlier [16]. The chip for cultivating neuronal cells consists of two chambers (length — 2.7 mm, width — 0.5 mm) connected by 8 microchannels 0.8 mm long. The chips were aligned with pre-cleaned MEA that contained 60 TiN electrodes with a diameter of 30 μm each and a distance of 200 μm between them (Multi Channel Systems, Germany). The latter procedure was performed using a stereotactic microscope to ensure that the microelectrodes were precisely placed in the microchannels and chambers (Figure 1).

### Cell culture.

 Neuronal cells were isolated from the hippocampus of mouse embryos (E18) and placed in the chambers of PDMS chips aligned with the microelectrode arrays; the initial cell density was around 7000–9000 cells/mm^2^ [17]. Euthanasia was performed by dislocating the cervical spine in accordance with the protocol approved by the Bioethics Committee of the National Research Lobachevsky State University of Nizhny Novgorod (Russia). Additional regulations included Order No.199n “On Approval of the Rules of Good Laboratory Practice” (Russia, 2016) and Directive 2010/63/EU of the European Parliament and the Council of the European Union (22.09.2010) on the protection of animals used for scientific purposes. The cells were grown in Gibco 21103-049 culture medium (Invitrogen, USA) with B27 bioactive supplement (Gibco 17504-044, Invitrogen), glutamine (Thermo Fisher Scientific 25030024, Invitrogen), fetal calf serum (Gibco A3160801, Invitrogen), and gentamicin (PanReac AppliChem A1492,0005, AppliChem, Germany) under 37°C, 100% humidity, and 5% CO_2_ in an incubator (SHELLAB 3552-2, Sheldon Manufacturing, Inc., USA). Half of the medium in the chip was refreshed every 2 days. On days 2–4 after cell planting, their axons grew into the microchannels of the microfluidic chip. An Axio Observer A1 inverted microscope (Carl Zeiss, Germany) was used to assess the axon growth. Measurements of axonal growth in the microchannels were started from day 2. The growing axons reached the end of the 800 μm microchannel on days 5–7 *in vitro*. The experiments with the cell cultures began on week 3 of *in vitro* cell growth [18].

### Electrical stimulation and bioelectric response measurement.

 The electrophysiological activity of cultured neurons was recorded using a USB-MEA120-System (Multi Channel Systems) instrument at a sampling rate of 20 kHz. Electrical stimulation of cells was performed using a sequence of 30 biphasic impulses (amplitude 800 mV, duration 260 μs per phase, the first phase was positive) with an inter-stimulus interval of 3 s. The stimuli were applied to 8 electrodes located in the microchannels.

In this study, only directly evoked spikes propagating through the axons of stimulated cells were recorded; synaptic responses were blocked by adding the AMPA receptor antagonist CNQX (Tocris Bioscience, USA) at a concentration of 10 μM and the inhibitor of N-methyl-D-aspartate (NMDA) receptors (R)-CPP (Tocris Bioscience) at a concentration of 10 μM.

### Optical stimulation.

 In order to detect the signals caused by optical stimulation applied to the culture of neuronal cells, we developed a system consisting of two modules. The first module performed temporal modulation of the laser beam; the second module reshaped the beam and focused it on the microelectrode array containing the cell culture. The first module consisted of a CW fiber laser, a spherical converging lens system, and a shutter; the second module consisted of a condenser, an MEA with a microfluidic chip, and a multichannel recording system. The two modules were connected by a transport fiber (DILAS Diodenlaser, Germany) (Figure 1 (c)).

In this study, we used two lasers as sources of electromagnetic radiation.

1. A CW holmium fiber laser (developed in the Lobachevsky University) generated radiation at a wavelength of 2095 nm. A series of impulses with durations of 1, 3, or 6 ms were used. The intervals between the stimuli were 3 s or 5 s; the radiation power varied from 0.1 W to 0.3 W. Continuous radiation was converted to the pulsed one using a manually operated mechanical shutter.
2. A CW thulium fiber Tm-laser LMT-30A-01 (IRE-Polyus, Russia) generated radiation at a wavelength of 1907 nm. Stimulation was carried out with pulses of 6 and 10 ms with an interval of 5 s. The radiation power was varied from 0.2 to 0.5 W.

A computer-controlled shutter (Avesta-Project, Russia) was used to create pulsed radiation. The pulse-periodic optical signal resulted from the shutter was accompanied by a TTL signal generated by the device at the moment of opening. The TTL signal was fed to a stimulation and registration system (Multi Channel Systems). The time delay between the delivery of a TTL signal and a laser impulse was about 10 ms.

At the output from the fiber, the radiation was collimated by a lens into a beam of 5 mm in diameter and then passed to the shutter. Thus, the continuous radiation was converted into pulses of a given duration. After the shutter, there was a lens that focused the radiation onto the input of a 5 m long transport fiber, through which the radiation entered the second module. In the second module, the fiber output was fixed in a condenser consisting of two collecting lenses with focal lengths of 50 and 40 mm, and a cylindrical lens with a focal length of 25 mm.

The microelectrode array with a cell culture in a microfluidic chip was located in a horizontally fixed connector of a multichannel signal recording system. The condenser was located vertically under the connector and focused the radiation onto the MEA through an opening in the bottom surface of the connector. The radiation power supplied to the MEA through a saline solution, as well as the power absorbed by the medium was determined using a FieldMaxII-TO laser power meter (Coherent, USA) in a continuous mode with no shutter.

In order to ensure sterility and optimal conditions for maintaining cell viability, a two-component PDMS cover structure was attached to the MEA to provide the flow of air with 5% CO_2_.

### Data processing.

 To analyze the responses evoked by electrical stimulation, the number of spikes detected by the electrodes in the microchannels was plotted against the time within a 50 ms window. Using graphs of that type, we compared the spikes obtained before and after blocking the synaptic responses. Those recorded after the blocking step represented the axonal responses and were further investigated using optical stimulation.

For responses evoked by optical stimulation, the number of induced spikes (from 5 ms to 50 ms) was plotted against the time within a 50 ms window. Two artifacts caused by optical stimulation were recorded at delays of 10 ms and 16–20 ms.

The probability of evoking a response by optical radiation was assessed for each of 24 electrodes installed in the microchannels: that was the number of stimuli needed to cause at least one spike (detected by one electrode) divided by the total number of stimuli applied. Then the mean probability values were determined for all 24 electrodes.

Experiments were carried out to determine the probabilities of responses evoked at different power levels of optical radiation (3 experiments, 2 MEA) and to assess the impact of radiation localization (1 experiment) and duration (1 experiment) on this probability.

### Statistical analysis.

 Comparison of the mean probability values at different radiation power levels was performed using the Wilcoxon T-test. The results are presented as M±SD, where M is the arithmetic mean, SD is the standard deviation. P≤0.05 is taken as the critical level of significance.

## Results and Discussion

### Study of axons grown in the microchannels.

 On day 2 of cell cultivation, the cell processes began to grow into the microchannels; on day 7, they reached the adjacent chamber. The microchannels were 800 μm long, which made it possible to separate axons from dendrites; the latter was about 400 μm long, i.e., typical for the hippocampus [19]. The small cross-section of the microchannels prevented the cell body from penetrating into the channels. In general, this method ensured the predominant presence of axons in the channels, which was also confirmed by the recorded signals.

Starting from day 15 of *in vitro* culture development, we were able to record the spontaneous activity of neurons in the chamber: these were series of spikes separated by intervals of 2–10 s. The spikes propagated along the axons growing in the microchannels. The time sequence of spontaneous spikes on adjacent electrodes was determined and the velocity of spike propagation along the axons was estimated. In our experiments, that was 361±141 mm/s (average of 7 channels), which corroborated with the reported propagation velocity of action potentials along unmyelinated axons in hippocampal slices — 380 mm/s [20].

### Action potentials evoked in axons by electrical stimuli.

**Figure 2:**
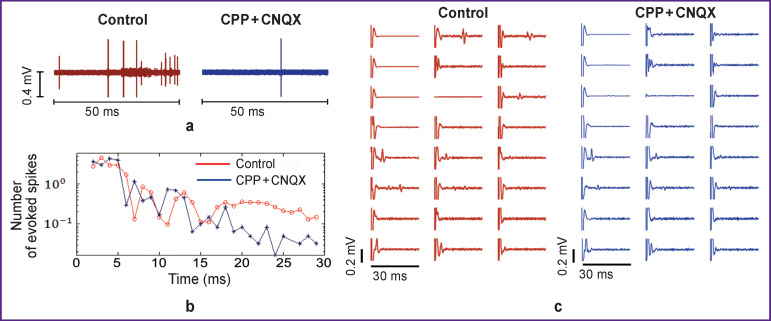
Bioelectrical activity recorded in axons grown into microchannels before (*left*) and after (*right*) the addition of synaptic transmission blockers — CPP — 10 μM and CNQX — 10 μM: (a) spontaneous activity recorded by one electrode; the number of evoked spikes decreases after the application of synaptic transmission blockers; (b) histogram of evoked activity recorded by all electrodes in the microchannels in 2–30 ms time window after an electrical stimulus is applied; the red line corresponds to the control conditions, the blue line corresponds to the conditions in the presence of synaptic transmission blockers; the direct axonal response is triggered at delays less than 15 ms and persists after adding the blockers; the synaptic response arising at time delays greater than 15 ms is absent when the blockers are added; (c) activity evoked by low-frequency electrical stimulation recorded in 8 microchannels; after the addition of the blockers, only direct responses propagating along axons are recorded

 After 2 weeks of neuronal cell cultivation, low-frequency stimuli were applied to axons using the electrodes located at the microchannels. Each stimulus elicited an action potential that propagated along the axons. These responses were triggered by changes in the axonal membrane potentials, and also by spikes evoked through synaptic contacts with other neurons. To study the axonal responses only, we blocked the excitatory receptors that mediated the synaptic transmission in the neuronal culture (Figure 2). The axonal response was detected after a delay of 15 ms (Figure 2 (a)).

### Action potentials evoked in axons by optical stimuli.

**Figure 3:**
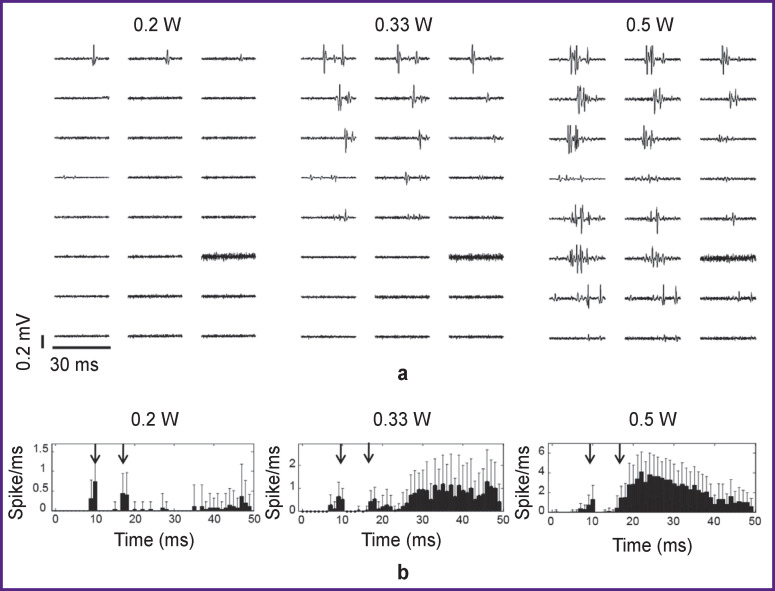
Bioelectric activity caused by optical stimulation as recorded in axons grown in the microchannels: (a) activity evoked by optical stimulation recorded in 8 microchannels at different radiation powers; an increase in the radiation power leads to an increase in the number of evoked spikes; (b) histograms of evoked activity recorded by all electrodes in the microchannels in a time window of 50 ms after an electrical stimulus is applied; the arrows indicate the optical stimulation artifacts that appear at the beginning and end of the pulse

 For optical stimulation, we selected those neuronal cultures where electrical stimulation evoked the axonal response. Optical stimulation by holmium laser pulses with a wavelength of 2095 nm and a radiation power of 0.1–0.3 W failed to induce any action potentials in the cultures. An artifact was recorded on the electrodes located in the area of laser illumination. During optical stimulation with a thulium laser at a wavelength of 1907 nm, two stimulation artifacts were recorded on the electrodes in the illumination region. The first artifact appeared at a delay equal to the delay between the TTL signal and the optical stimulus; the second one was detected at a delay equal to the duration of the optical pulse relative to the first artifact. The optically-induced action potentials were measured in axons along the microchannels. Notably, action potentials were not detected by the electrodes located in the cell culture chamber. The responses began to appear at a radiation power exceeding 0.2 W (Figure 3 (a)). Increasing the radiation power increased the occurrence rate of the responding spike (Figure 3 (b)) and the number of microchannels where the responses were detected (from 1 to 8). The occurrence rates of action potentials were 10±9% at a power of 0.2 W, 33±6% at 0.33 W, and 59±15% at 0.5 W (3 experiments, 2 MEA). Differences between these percentages were significant for 0.2 vs 0.33 W as well as for 0.33 vs 0.5 W (Wilcoxon T-test, p<0.05).

Next, we investigated the relations between the duration of optical laser pulses and the occurrence rate of axon responses. To this end, we determined the minimum radiation powers required to evoke action potentials in the axons. For pulses of 6 ms long, this value was 0.2 W and for pulses of 10 ms — 0.5 W. The evoked spikes were observed in 8 channels when stimulated with laser pulses of 6 ms long (the occurrence rate was 75±3% for 24 electrodes) and in only one channel — when stimulated with pulses of 10 ms (the occurrence rate was 5±13% for 24 electrodes). In further experiments, the pulse duration was chosen at 6 ms.

Then, we investigated whether the location of the radiation beam affected the occurrence of responding spikes detected in the axons. Accordingly, we compared the axon response rates between two scenarios: the radiation was focused on the culture chamber and the radiation was focused on the microchannels with the axons. When neuronal cells were irradiated, spikes in axons appeared in ≥3 microchannels, and the number of these channels increased with increasing radiation power thus reaching 6 at 0.5 W (the occurrence rate 58±3%). By comparison, stimulation of axons in the microchannels elicited responses only in 2 microchannels at a radiation power of 0.5 W (the occurrence rate 22±2%).

To elucidate the nature of the responses evoked by optical stimulation, we added the sodium channel blocker tetrodotoxin to the culture; this inhibitor was supposed to block neuronal spike transmission. Indeed, under these conditions, we were unable to detect any spike evoked by optical stimulation.

Thus, a new method for the induction of action potentials by pulsed IR irradiation of neuronal cell cultures has been developed; these action potentials propagate through axons grown in microchannels of a microfluidic chip. Earlier [15], it was shown that in homogeneous cell cultures cultivated on MEA, a laser pulse caused an action potential recorded only by one neighboring electrode, presumably in the axon of one neuron. In contrast, we used MEA electrodes located outside the irradiation area to record action potentials in axons grown into microchannels. When a laser pulse was applied to the cell bodies, the responding action potential could be observed along the channel every 200 μm at a distance of up to 800 μm. In the presence of a sodium channel blocker, no action potential could be evoked in the neuronal culture, which confirms the neuronal nature of the evoked response.

The radiation power (0.2–0.5 W) and wavelength (1907 nm) of the IR pulses used in this study were similar to those used by others with retinal ganglion cells and vestibular ganglion cells (0.2 W; 1875 nm), as well as cultures of cortical neurons (0.6 W; 1940 nm) [12, 15]. We found a positive correlation between the radiation power and the probability of a response evoked in axons (3 experiments, 2 MEA) (see Figure 3). In this case, the probability of a response evoked by optical stimulation was higher with a shorter duration of the optical stimulus (6 ms as compared to 10 ms). We also found that the response action potentials occurred more frequently when the radiation pulse was applied to the cell somas rather than to the axons.

## Conclusion

A method for non-invasive induction of spikes in neuronal culture using optical stimulation has been developed and successfully tested. The technology included a microfluidic chip that provided for the growth of neuronal axons and an optical stimulation unit combined with a multichannel bioelectric recording system. The new method allows to non-invasively induce and study action potentials propagating along the axons at a distance of up to 800 microns over days or months. The method can be used both in fundamental research to study the brain signaling system and in the development of a non-invasive neural-interface.

## References

[1] Shapiro M.G., Homma K., Villarreal S., Richter C.P., Bezanilla F (2012). Infrared light excites cells by changing their electrical capacitance.. Nat Commun.

[2] Lumbreras V., Bas E., Gupta C., Rajguru S.M (2014). Pulsed infrared radiation excites cultured neonatal spiral and vestibular ganglion neurons by modulating mitochondrial calcium cycling.. J Neurophysiol.

[3] Cayce J.M., Friedman R.M., Jansen E.D., Mahavaden-Jansen A., Roe A.W (2011). Pulsed infrared light alters neural activity in rat somatosensory cortex in vivo.. Neuroimage.

[4] Cayce J.M., Friedman R.M., Chen G., Jansen E.D., Mahavaden-Jansen A., Roe A.W (2014). Infrared neural stimulation of primary visual cortex in non-human primates.. Neuroimage.

[5] Wells J., Kao C., Jansen E.D., Konrad P., Mahadevan-Jansen A (2005). Application of infrared light for in vivo neural stimulation.. J Biomed Opt.

[6] Wells J., Kao C., Konrad P., Milner T., Kim J., Mahadevan-Jansen A., Jansen E.D (2007). Biophysical mechanisms of transient optical stimulation of peripheral nerve.. Biophys J.

[7] McCaughey R.G., Chlebicki C., Wong B.J.F. (2010). Novel wavelengths for laser nerve stimulation.. Lasers Surg Med.

[8] Izzo A.D., Richter C.P., Jansen E.D., Walsh J.T. Jr. (2006). Laser stimulation of the auditory nerve.. Lasers Surg Med.

[9] Jenkins M.W., Duke A.R., Gu S., Chiel H.J., Fujioka H., Watanabe M., Jansen E.D., Rollins A.M (2010). Optical pacing of the embryonic heart.. Nat Photonics.

[10] Wang Y.T., Rollins A.M., Jenkins M.W (2016). Infrared inhibition of embryonic hearts.. J Biomed Opt.

[11] Richter C.P., Matic A.I., Wells J.D., Jansen E.D., Walsh J.T. Jr. (2011). Neural stimulation with optical radiation.. Laser Photon Rev.

[12] Albert E.S., Bec J.M., Desmadryl G., Chekroud K., Travo C., Gaboyard S., Bardin F., Marc I., Dumas M., Lenaers G., Hamel C., Muller A., Chabbert C (2012). TRPV4 channels mediate the infrared laser-evoked response in sensory neurons.. J Neurophysiol.

[13] Fekete Z., Horváth Á.C., Zátonyi A. (2020). Infrared neuromodulation: a neuroengineering perspective.. J Neural Eng.

[14] Xia Q., Nyberg T (2019). Inhibition of cortical neural networks using infrared laser.. J Biophotonics.

[15] Xia Q.L., Wang M.Q., Jiang B., Hu N., Wu X.Y., Hou W.S., Nyberg T (2019). Infrared laser pulses excite action potentials in primary cortex neurons in vitro.. Annu Int Conf IEEE Eng Med Biol Soc.

[16] Gladkov A., Pigareva Y., Kutyina D., Kolpakov V., Bukatin A., Mukhina I., Kazantsev V., Pimashkin A (2017). Design of cultured neuron networks in vitro with predefined connectivity using asymmetric microfluidic channels.. Sci Rep.

[17] Gladkov A.A., Kolpakov V.N., Pigareva Y.I., Bukatin A.S., Kazantsev V.B., Mukhina I.V., Pimashkin A.S (2017). Study of stimulus-induced plasticity in neural networks cultured in microfluidic chips.. Sovremennye tehnologii v medicine.

[18] Pimashkin A., Gladkov A., Mukhina I., Kazantsev V (2013). Adaptive enhancement of learning protocol in hippocampal cultured networks grown on multielectrode arrays.. Front Neural Circuits.

[19] Taylor A.M., Blurton-Jones M., Rhee S.W., Cribbs D.H., Cotman C.W., Jeon N.L (2005). A microfluidic culture platform for CNS axonal injury, regeneration and transport.. Nat Methods.

[20] Meeks J.P., Mennerick S (2007). Action potential initiation and propagation in CA3 pyramidal axons.. J Neurophysiol.

